# Phonon Surface Scattering and Thermal Energy Distribution in Superlattices

**DOI:** 10.1038/s41598-017-05631-3

**Published:** 2017-07-17

**Authors:** Kartik Kothari, Martin Maldovan

**Affiliations:** 1 School of Physics, Georgia Institute of Technology, Atlanta, Georgia 30332 USA; 2 School of Chemical & Biomolecular Engineering, Georgia Institute of Technology, Atlanta, Georgia 30332 USA

## Abstract

Thermal transport at small length scales has attracted significant attention in recent years and various experimental and theoretical methods have been developed to establish the reduced thermal conductivity. The fundamental understanding of how phonons move and the physical mechanisms behind nanoscale thermal transport, however, remains poorly understood. Here we move beyond thermal conductivity calculations and provide a rigorous and comprehensive physical description of thermal phonon transport in superlattices by solving the Boltzmann transport equation and using the Beckman-Kirchhoff surface scattering theory with shadowing to precisely describe phonon-surface interactions. We show that thermal transport in superlattices can be divided in two different heat transport modes having different physical properties at small length scales: layer-restricted and extended heat modes. We study how interface conditions, periodicity, and composition can be used to manipulate the distribution of thermal energy flow among such layer-restricted and extended heat modes. From predicted frequency and mean free path spectra of superlattices, we also investigate the existence of wave effects. The results and insights in this paper advance the fundamental understanding of heat transport in superlattices and the prospects of rationally designing thermal systems with tailored phonon transport properties.

## Introduction

**Figure 1:**
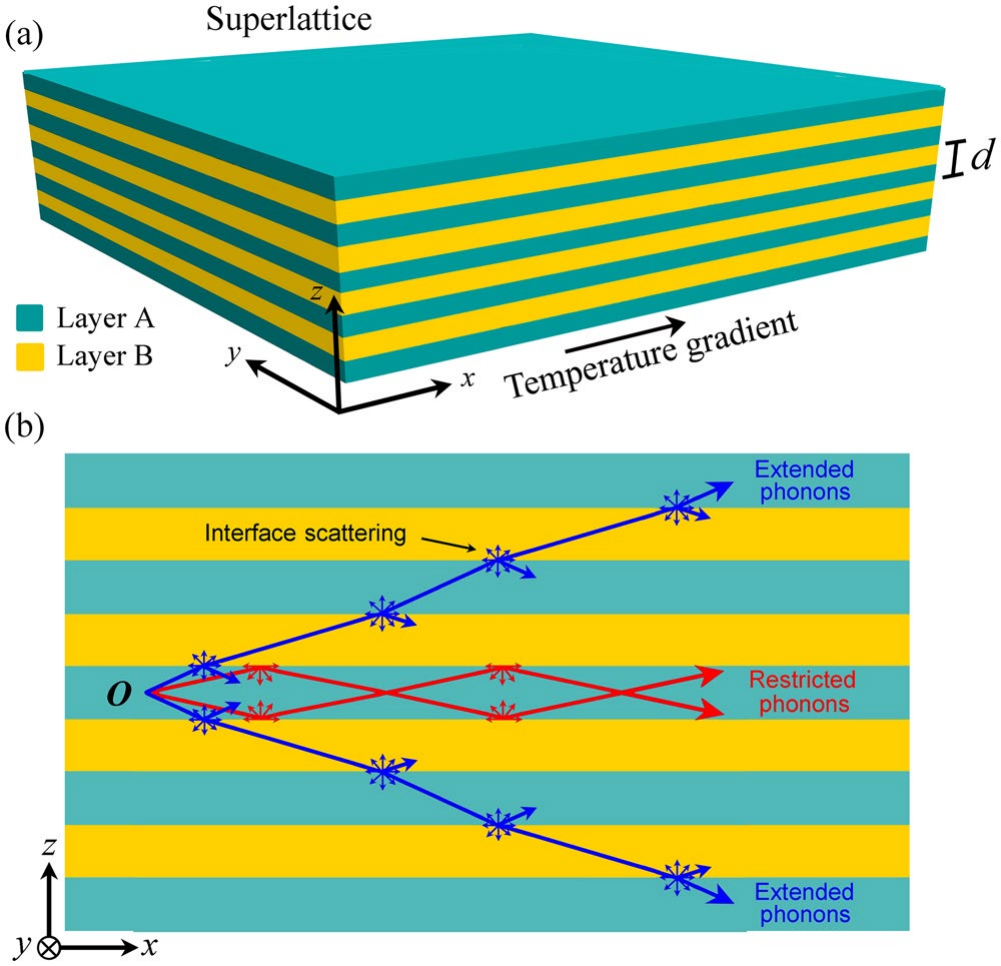
Schematic representation of phonon transport in superlattices. (**a**) A superlattice made of materials *A* and *B* in which a temperature gradient is applied along the *x* direction. (**b**) Phonons originating at point *O* within the superlattice can travel within a single layered, i.e. layer-restricted (red) or they can cross multiple interfaces and propagate in different layers, i.e. extended (blue).

The understanding and manipulation of thermal transport at reduced length scales is crucial to improve the efficiency of energy materials, modern day lasers, microelectronics, and nanofabricated devices^1, 2^. The independent control of heat and charge carriers (i.e. phonons and electrons) enables efficient thermoelectric energy conversion since a reduction in thermal conductivity, while maintaining electrical conductivity, results in the enhancement of the thermoelectric figure of merit *ZT* = *S* ^2^ *σT*/*κ*, where *S* is the Seebeck coefficient, *T* the temperature *σ* the electrical conductivity, and *κ* the thermal conductivity^3^. In addition, ineffective heat dissipation is currently a limitation on efficiency of nano and micro optoelectronic devices. The most consequential mechanism affecting thermal transport at the nanoscale is the interaction of phonons with interfaces and boundaries^4^. Ubiquitous systems wherein these interactions significantly modify thermal transport are layered structures such as superlattices. Superlattices are periodic systems constituted of two materials in which the periodicity induces different physical properties than those of the constituents. Their ease of manufacture and tunability has made superlattices a standard platform for semiconductor devices with applications in energy conversion^5^, electronics^6^, photonics^7^, and phononics^8^. The thermal conductivity of superlattices is considerably affected by the scattering of phonons at interfaces and thus differs from the conductivity of a layered system of the same materials at the bulk/macro scale. Phonons with relatively short wavelengths are scattered diffusely by the superlattice interfaces and they contribute negligibly to the in-plane thermal conductivity after scattering (Fig. 1)^4^. In contrast, phonons with large wavelengths are scattered specularly and allowed to transmit and reflect, contributing to heat flux after scattering^9^. Despite significant advances in the last decades, current approaches are not able to determine how thermal phonons are transported in realistic superlattices and to establish the amount of phonons that are diffusely or specularly scattered at interfaces. This is in part due to the lack of rigorous physical descriptions of phonon surface scattering phenomena, which fail to include realistic and measurable surface features (e.g. surface roughness and correlation lengths). Importantly, this limitation has prevented the understanding and control of thermal transport processes in superlattices and the design of novel thermal transport phenomena such as coherent interference and heat mirrors^10^.

Theoretical methods to study thermal transport in superlattices can be divided into atomistic (e.g. molecular dynamics MD, density-functional-theory DFT) and continuum (e.g. Boltzmann transport) approaches each having its own advantages and drawbacks. MD techniques provide useful insights on the dependence of thermal conductivity on different physical variables and first-principle approaches allow to study the thermal conductivity based on DFT techniques. The detailed nanostructure surface features and their impact on thermal conductivity, however, are difficult to be incorporated. On the other hand, Boltzmann Transport Equation BTE approaches require the bulk phonon mean-free-path as input, which needs to be obtained using other methods (DFT) or fitting parameters. Once the bulk phonon mean-free-path is known, the thermal conductivity of multiple nanostructures (including surface characteristics) can be readily obtained without using fitting parameters. Another contrast between atomistic and continuous approaches is the length scale range; atomistic simulations are computationally intensive at large scales preventing the prediction of nano-to-bulk thermal properties, while the BTE allows bulk-to-nanoscale predictions but requires incorporation of wave effects (e.g. coherent interference and confinement) at very small length scales.

Using molecular dynamics, Daly *et al*.^11^ studied thermal transport in a simplified model for GaAs/AlAs superlattices. They found that for superlattices having smooth interfaces, the cross-plane thermal conductivity shows a minimum as a function of the superlattice period. The existence of such minimum was previously predicted using wave theory and indicates the presence of phonon wave effects^12^. In contrast, for superlattices with interfacial species mixing, the thermal conductivity increased with period length as observed in experiments^13, 14^. Trends in cross-plane thermal conductivity in superlattices were also investigated by Chen *et al*.^15^. They found that a minimum in thermal conductivity occurred if the mean free path MFP is close to or larger than the period and the lattice constants are similar. From MD simulations^11–19^, it is clear that a minimum in thermal conductivity exists if the superlattice interfaces are perfect and they vanish for interfaces with species mixing. The precise determination of phonon wave effects and their relation to surface features (i.e. roughness and correlation lengths) is however difficult to obtain with current MD models.

Using first-principles, Garg *et al*.^20^ analyzed the thermal conductivity of Si/Ge superlattices with relatively smooth surfaces and found that, for increasing period, the in-plane conductivity increases monotonically while a minimum was observed for the cross-plane configuration. More recently, the cross-plane thermal conductivity in Si/Ge superlattices via ab initio calculations was investigated and compared with experiments by Chen *et al*.^21^, and found that surface segregation and intermixing of atoms may further reduce thermal conduction. In addition, Garg *et al*.^22^ predicted using DFT that short-period superlattices with perfect interfaces could have high conductivity due to reduced availability of scattering channels. Tian *et al*.^23^ also calculated the cross-plane thermal conductivity using atomistic Green’s functions and found an optimum period length which minimizes the thermal conductivity. Detailed surface features and their effects are difficult to include in first-principle approaches while Green’s function methods approximate thermal conduction by considering ballistic transport.

In addition to atomistic models, the Boltzmann transport equation (BTE) was employed by Chen^24–26^ to study in-plane and cross-plane thermal conductivity in GaAs/AlAs and Si/Ge superlattices, respectively. It was found that accounting for frequency dependent internal scattering and partially diffuse and partially specular interfaces was fundamental to obtain agreement between theory and experiments. Liu *et al*.^27^ evaluated the cross-plane thermal conductivity of Si/SiGe superlattices theoretically using BTE and experimentally by the 3ω technique. The thermal conductivity was found to decrease with decreasing ratio of layer thickness, specularity and period length. These BTE models consider frequency independent phonon-surface interactions and the interface specularity is thus incorporated as an empirically adjusted parameter. More recently, Aksamija *et al*.^28^ proposed a simplified BTE model to calculate the thermal conductivity of Si_x_Ge_1−x_/Si_y_Ge_1−y_ superlattices using a surface specularity parameter dependent on wavevector and interface roughness. The model was employed to obtain the anisotropy, temperature, and period variation of the thermal conductivity. Building up on this model, Mei *et al*.^29^ recently calculated the in-plane and cross-plane thermal conductivities of III-V superlattices. Being an improvement with respect to constant specularity models, the proposed BTE approach does not consider phonon reflections and transmissions at the interfaces, which is the fundamental principle that give rise to phonon coherent interference and significantly influence the superlattice thermal conductivity.

Experimental measurements of superlattice thermal conductivity have shown a variety of trends. Lee *et al*.^14^ showed that the cross-plane thermal conductivity of Si-Ge superlattices increased with increasing period length and suddenly decreased due to formation of dislocations. On the other hand, Capinski *et al*.^30^ measured the cross-plane thermal conductivity of GaAs/AlAs superlattices and found that it increases with increasing period length. Furthermore, Huxtable *et al*.^31^ observed that the thermal conductivity of Si/SiGe alloy superlattices increased with increasing period while that of Si_*x*_Ge_1−*x*_/Si_*y*_Ge_1−*y*_ superlattices was weakly dependent on period. More recently, experiments on epitaxial oxide superlattices by Ravichandran *et al*.^32^ exhibited a minimum in the thermal conductivity as a function of period length, thereby demarcating a transition from coherent to incoherent phonon surface scattering. In terms of the temperature dependence, experiments show that the thermal conductivity of Si/Ge superlattices increase up to a temperature of ~200 K, after which it remains fairly constant^14, 31, 33–35^.

Due to increased surface-to-volume ratios, a critical mechanism determining the thermal conductivity of nanostructures is phonon-surface scattering. Current theoretical approaches however do not incorporate a rigorous treatment of surface scattering phenomena that allows to describe realistic phonon-surface interactions and account for relevant physical variables such as phonon momentum and angle of incidence as well as surface roughness and correlation lengths. A fundamental understanding of superlattice thermal transport requires not only to establish the thermal conductivity but also to elucidate how phonons move within the system. Developing such deep understanding necessitates the establishment of the amount of phonons that are specularly and diffusely scattered at the interfaces, the amount of heat that is channeled within a single superlattice layer, and the amount of heat that is transported across multiple layers. Such a thorough insight can only be achieved by theoretical approaches that accurately couple phonon transport within the layered materials and phonon scattering phenomena at the interfaces. The approaches would allow to design and manipulate heat transport in superlattices and exploit their unique thermal transport properties such as layer-guided thermal transport and the coherent interference of thermal phonons. Here, we provide a rigorous thermal transport analysis using the Boltzmann Transport Equation to predict and study various physical mechanisms behind thermal transport processes in superlattices, including phonon coupling between layers, reflection and transmission conditions, and specular and diffuse scattering of thermal energy at interfaces. We extend the electromagnetic wave scattering theory developed for rough surfaces by Beckmann and Spizzichino^36^ to accurately model reflection and transmission at rough interfaces as well as phonon coupling between the layers by accounting for all the relevant physical variables such as phonon properties (e.g. momentum, incident angle) and surface characteristics (roughness, correlation lengths, and shadowing)^37^. By directly calculating the reduction in phonon relaxation times due to surface scattering through the rigorous solution of the Boltzmann transport equation, our analysis moves beyond the Mattheissen rule approximation for modelling surface scattering. Importantly, using our model, we provide a detailed description of the amount of heat that is restricted within the constituent layers (layer-restricted phonons) and that which is specularly transmitted across interfaces and propagates across different layers (extended phonons). In addition, we present a thorough study of the heat spectrum which allows to predict the relative proportion of heat carried by phonons of different frequencies and mean-free-paths. We also show how various components of thermal transport (layer-restricted and extended) are dependent on period length, different surface conditions, and volume fraction of the constituents. Our predictions for variation of thermal conductivity with temperature show good agreement with experimental measurements. We also discuss the possibility of wave effects due to surface scattering for phonons that are restricted to propagate in individual layers and those that propagate among different layers in the superlattice.

## Results and Discussion

To obtain a precise physical description of phonon transport in superlattices it is important to understand first how thermal energy flow is spatially distributed within the system (Fig. 1b). Phonons originating at point *O* within a superlattice can follow different paths depending on their frequency and incident angle. Some phonons can reach an interface and undergo specular transmission into the adjacent layer and continue to propagate in the different layers of the superlattice. We call these “extended phonons”. Phonons can also reach an interface and undergo total reflection due to two different physical principles: total internal reflection or band structure mismatch (i.e. no accessible frequency on the adjacent layer). Since these phonons move within a single layer, they are called “layer–restricted phonons”. Note that if the reduced phonon MFP is less than the distance needed to reach an interface in the direction of its wavevector, the phonons will also be layer restricted. Our goal is to move beyond calculation of thermal conductivity and to establish how thermal phonons are transported within superlattices. To achieve this goal, we first predict the amount of heat carried by extended and layer-restricted phonons and provide insights on the effects of periodicity and surface scattering. As we discuss in the next sections, extended and layer-restricted phonons constitute different heat conduction modes with distinct physical properties that can give rise to different wave-interference phonon effects on thermal energy transport.

### Role of periodicity on thermal energy distribution among superlattice layers

**Figure 2:**
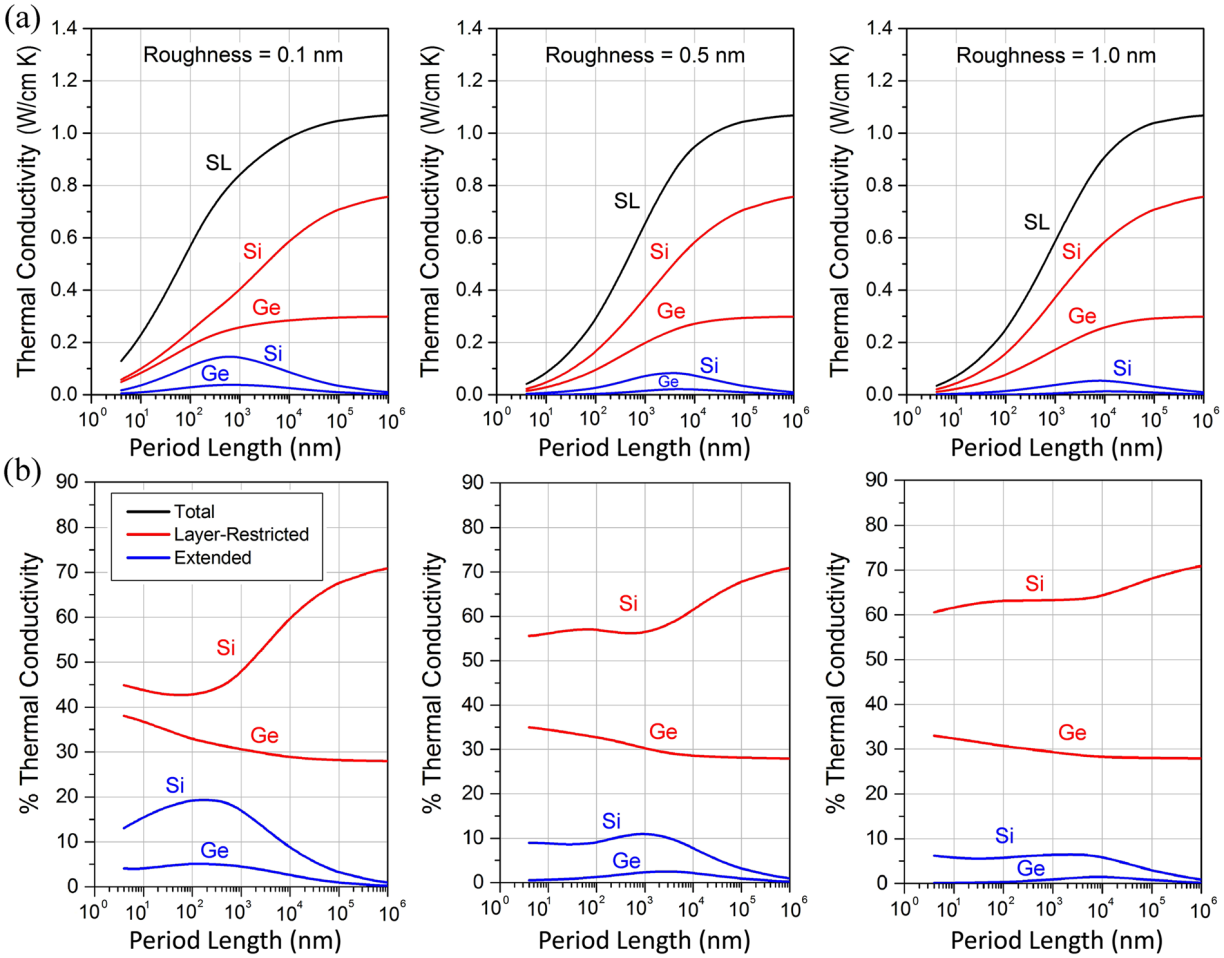
(**a**) Distribution of thermal energy carried by for layer-restricted phonons (red), extended phonons (blue), and total thermal conductivity (black) as a function of superlattice period length for surface roughness η = 0.1 nm, 0.5 nm, and 1.0 nm. (**b**) Variation of percentage contribution of extended and layer-restricted phonons in Si and Ge to the total superlattice thermal conductivity.

We show in Fig. 2 the effects of superlattice period on the thermal conductivity and energy distributions for Si/Ge superlattices under different interface conditions at room temperature. The different contributions to thermal conductivity arising from extended (e-Si, e-Ge) and layer-restricted (r-Si, r-Ge) phonons are shown separately with blue and red lines respectively while black lines show the total in-plane superlattice thermal conductivity κ_SL_. Note that e-Si refers to phonons that are extended and originating in Si, while e-Ge refers to extended phonons originating in Ge. Bulk phonon mean-free-paths and dispersion relations for Si and Ge are taken from existing values in the literature^38–41^. We observe that κ_SL_ decreases as the period length decreases, which is attributed to increased phonon interface scattering due to an increased interface density and the absence of wave effects (see Wave effects). Figure 2a quantitatively shows how κ_SL_ varies with period for different surface roughnesses (*η* = 0.1, 0.5, and 1 nm) at the interfaces. While κ_SL_ is significantly reduced for larger η values at small length scales, for large periods κ_SL_ saturates to the bulk value ${\kappa }_{SL}=0.5({\kappa }_{bulk-Si}+{\kappa }_{bulk-Ge})=0.5(1.56+0.60)=1.08\,W/\mathrm{cm}{\rm{.K}}$ independently of the surface condition. This trend confirms to the physical concept that at large periods, superlattices behave as bulk multilayers and *κ*_*SL*_ does not depend on interface features. We found in Fig. 2a that, with increasing period, the contribution of extended phonons (i.e. those moving across different layers) undergoes a maximum and then vanishes asymptotically. The increase in contribution is due to the reduced interface density as the period increases, whereas the decline is due to the fact that as period lengths approach bulk values, the number of incident phonons on the interfaces reduces since phonon MFPs become much smaller than the layer thicknesses. That is, due to the limited extent of the bulk phonon mean-free-paths there are no extended phonons for large superlattice periods. Contrarily, the contribution of layer-restricted phonons (i.e. those moving in a single layer) increases monotonically with increasing period lengths and, similarly to *κ*_*SL*_, reach the bulk values 0.5 *κ*_*bulk*−*Si*_ and 0.5 *κ*_*bulk*−*Ge*_ for large periods. This is consistent since at large periods phonons do not see the interfaces and move within a single layer while carrying the heat. In Fig. 2b, we also analyze the relative contributions of the components of conductivity, i.e. their percentage contribution to the total conductivity, as a function of the period length and for different surface conditions. We found that with increasing period length, the relative contribution of phonons restricted to Si shows a minimum (correlated to the maximum in e-Si) while that of those restricted to Ge decreases. The relative contributions of phonons extended across layers also exhibit maxima before tending to negligible amounts at large period lengths. The contribution of different phonon transport modes to the thermal conductivity is dependent on the superlattice period and surface conditions and, in particular, we found that there exist a specific period for which the amount of heat carried by extended phonons is maximized.

### Influence of superlattice volume fraction

**Figure 3:**
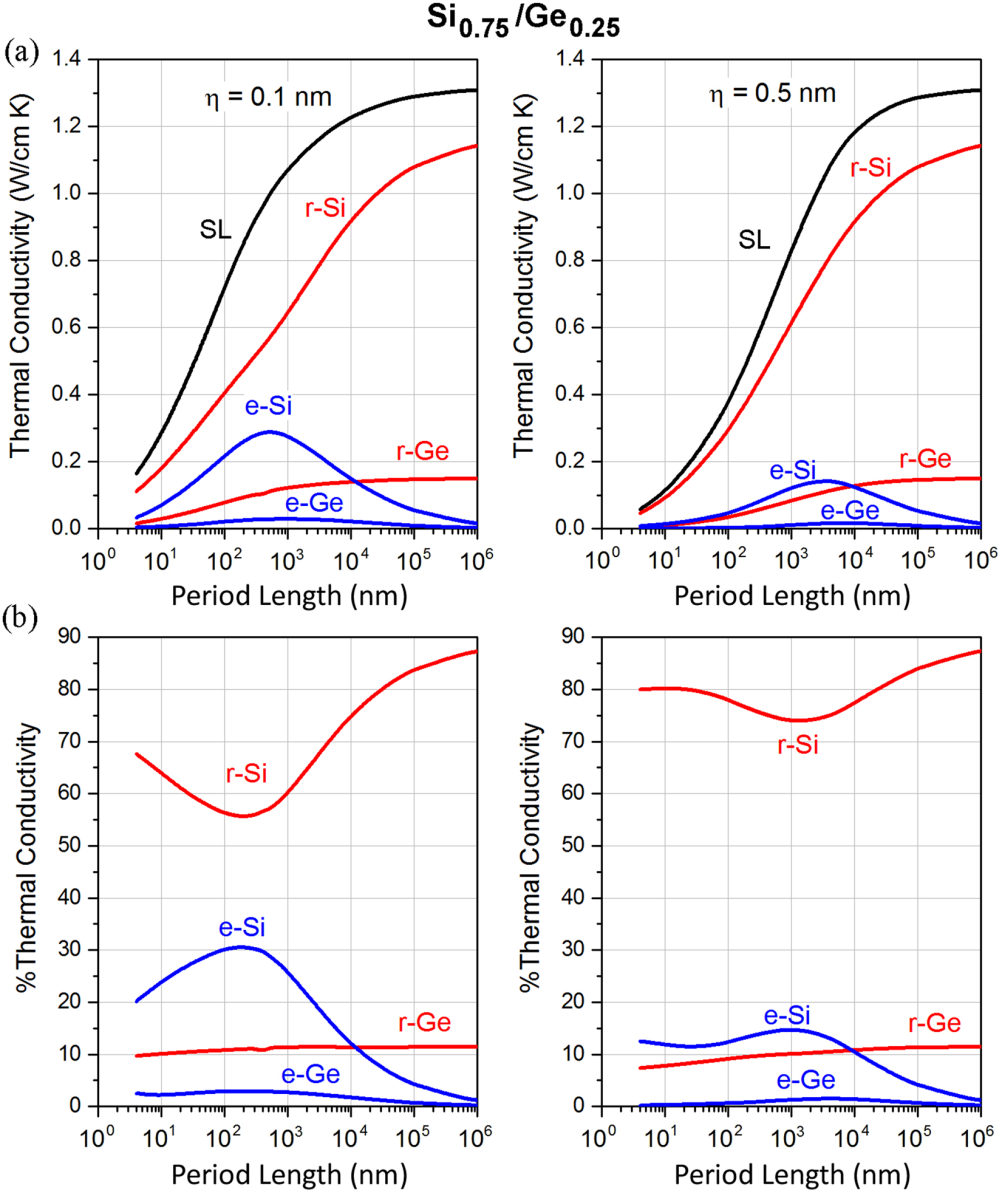
(**a**) Variation of thermal conductivity of layer-restricted and extended phonons as a function of period length for Si/Ge superlattices having volume fraction of *f*_Si_ = 0.75, with roughness values of 0.1 nm and 0.5 nm. (**b**) Relative contribution of extended and layer-restricted phonons in the corresponding superlattices.

**Figure 4:**
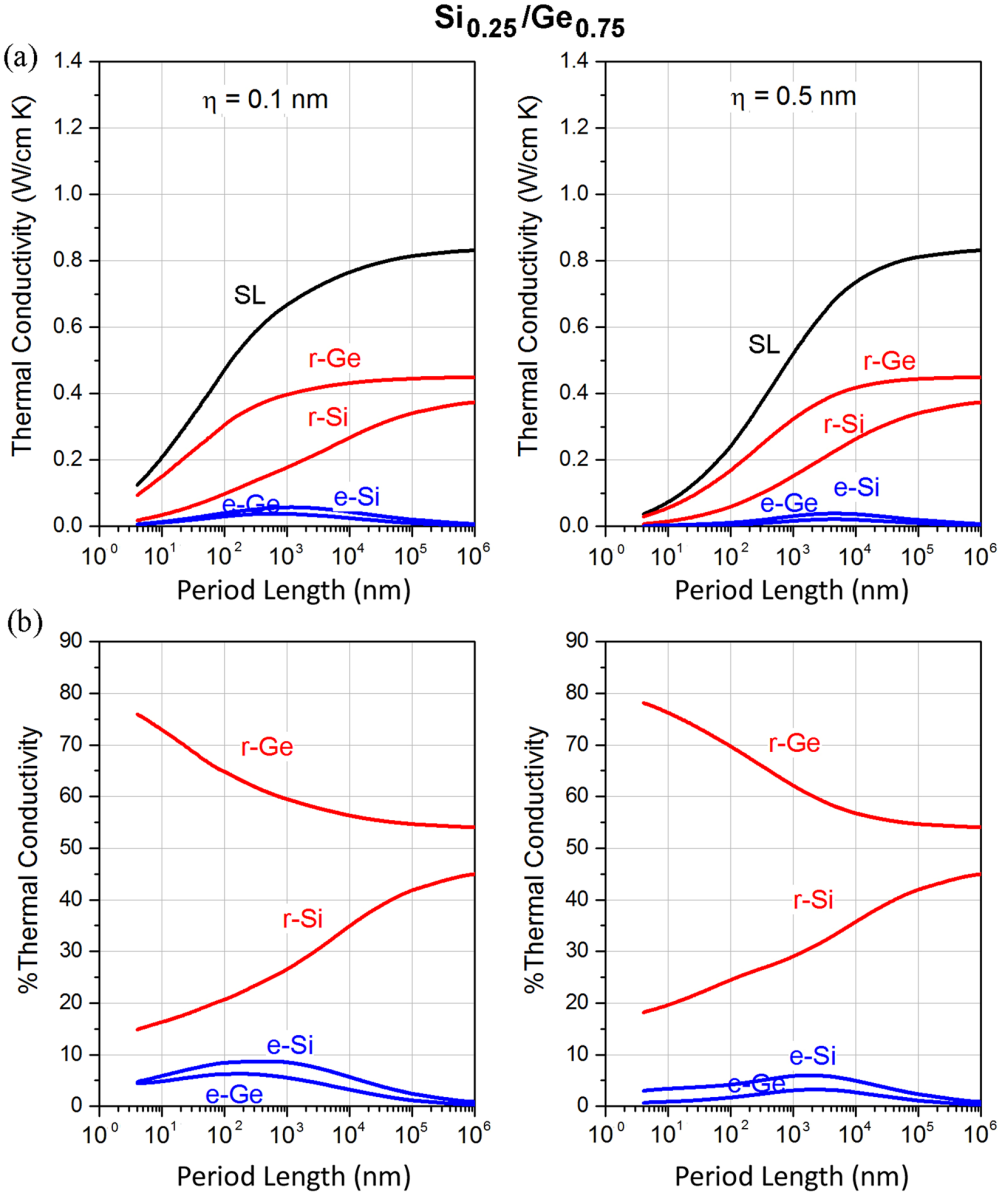
(**a**) Variation of thermal conductivities with period length for Si/Ge SLs, having volume fraction of *f*_Si_ = 0.25, with surface roughness η = 0.1 nm and η = 0.5 nm. (**b**) Relative contribution of extended and layer-restricted phonons.

Next we investigate the phonon energy distributions within the superlattices as a function of the volume fraction *f* of the constituent materials. We consider two vastly different SiGe superlattice conditions by choosing *f*_Si_ = 0.75 and *f*_Si_ = 0.25 and show in Figs 3 and 4 the variation of the thermal conductivity with period for different surface conditions. For a fixed period length, decreasing *f*_Si_ decreases the thermal conductivity κ_SL_, which is analogous to the bulk case, since *κ*_*bulk*−*Si*_ > *κ*_*bulk*−*Ge*_. For *f*_Si_ = 0.75 (Fig. 3), the contribution to conductivity of phonons restricted to Si is increased with respect to *f*_Si_ = 0.50 due to its higher volume fraction. Note that the contribution of extended phonons originating from Si and the maximum is also more pronounced than *f*_Si_ = 0.50 and for period lengths *d* < 10^4^ nm the contribution is larger than that of those restricted in Ge. For larger periods, however, a reversed behavior is observed due to the zero-limit contribution of extended phonons at large periods. In terms of relative contributions, phonons restricted to Si still exhibit a minima with increasing period length (Fig. 3b) which is in agreement with the trend found for *f*_Si_ = 0.50. The relative contributions of layer-restricted phonons in Ge are weakly dependent on the period length. On the other hand, a maximum is still found in the relative contribution of extended phonons from Si and Ge. For *f*_Si_ = 0.25 (Fig. 4), we observe that the contribution to conductivity of layer-restricted phonons in Ge is the largest (due to the large Ge volume fraction) followed by that of phonons restricted to Si and thereafter that of extended phonons originating from Si and Ge. This feature is maintained across all period length scales. In terms of relative contributions, the relative contribution of phonons restricted to Ge decreases while that of phonons restricted to Si increases with increasing period. Importantly, by comparing Figs 2, 3 and 4, we found that reducing the volume fraction of Si does not alter the existence of a maximum for extended phonons but the amount of heat carried by these phonons is significantly reduced. Note that all the aforementioned maxima and minima for the distribution of thermal energy are more pronounced in the case of small interface roughness as reduced interface scattering allows for larger mean free paths and thus a larger proportion of extended phonons.

### The impact of interface roughness

**Figure 5:**
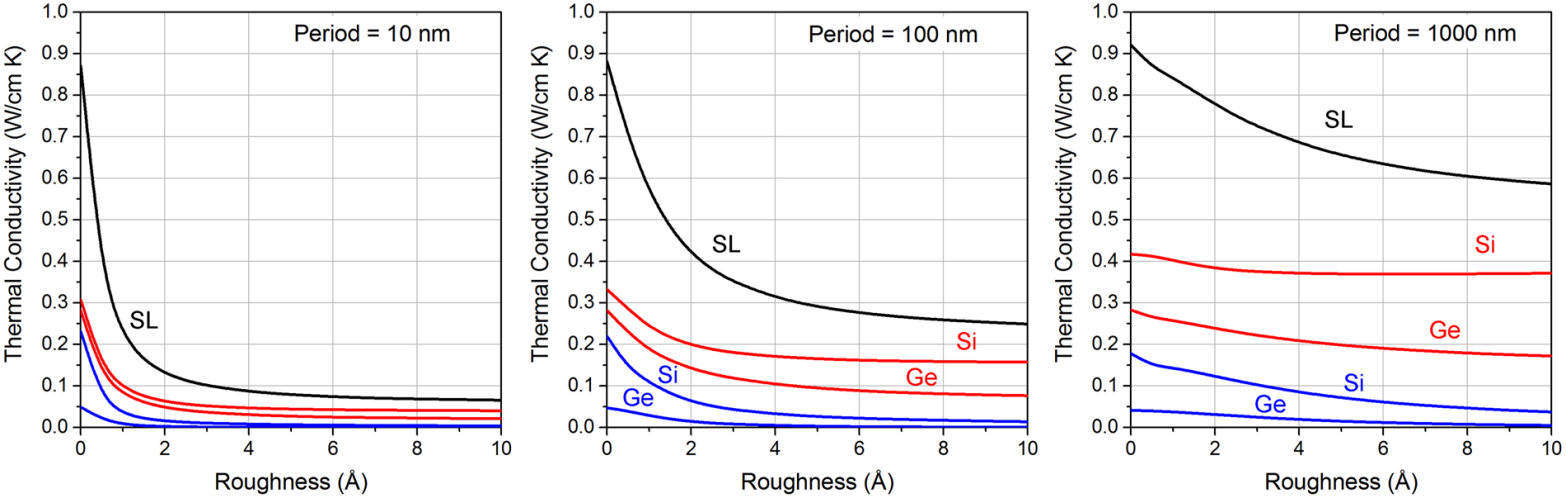
Effects of interface roughness on the amount of heat carried by layer-restricted and extended phonons in superlattices with periods *d* = 10 nm, 100 nm, and 1000 nm. Black, red and blue curves represent total, layer-restricted and extended components of conductivity respectively.

Since interface conditions are a critical aspect for superlattice thermal transport, we investigate in detail the variation of energy distribution and thermal conductivity with respect to surface roughness in superlattices *f*_Si_ = *f*_Ge_ = 0.50. Figure 5 shows κ_SL_ when the interface roughness is varied from *η* = 0 nm to 1 nm. We quantitatively show how κ_SL_ reduces and converges to a constant value as the roughness increases. This behavior is observed in restricted and extended components of the conductivity and across different period lengths. This is attributed to the fact that as we increase interface roughness, the fraction of phonons that are specularly scattered diminishes asymptotically. Very rough surfaces lead to complete diffusive scattering and that gives the lowest value of conductivity for a specific period length. On the other hand, we observe that κ_SL_ increases at *η* = 0. This is due to the absence of diffuse interface scattering in the superlattice. In this case, it is only the impedance mismatch which modulates the amount of heat carried in different constituent elements. Since impedance mismatch is non-vanishing in Si/Ge SLs, we note that κ_SL_ does not tend to bulk values at *η* = 0 in the quasi-ballistic regime.

From the previous discussions, it is clear that the amount of thermal energy carried by layer-restricted and extended phonons can be tailored by manipulating the structural features of the superlattice such as period, volume fraction, and surface roughness. This means that it is possible to rationally design superlattices with maximal or minimal proportions of heat carried by extended or layer-restricted phonons. These different heat transport processes can give rise to different physical properties and wave effects as we discuss later.

### Heat frequency spectrum in superlattices

**Figure 6:**
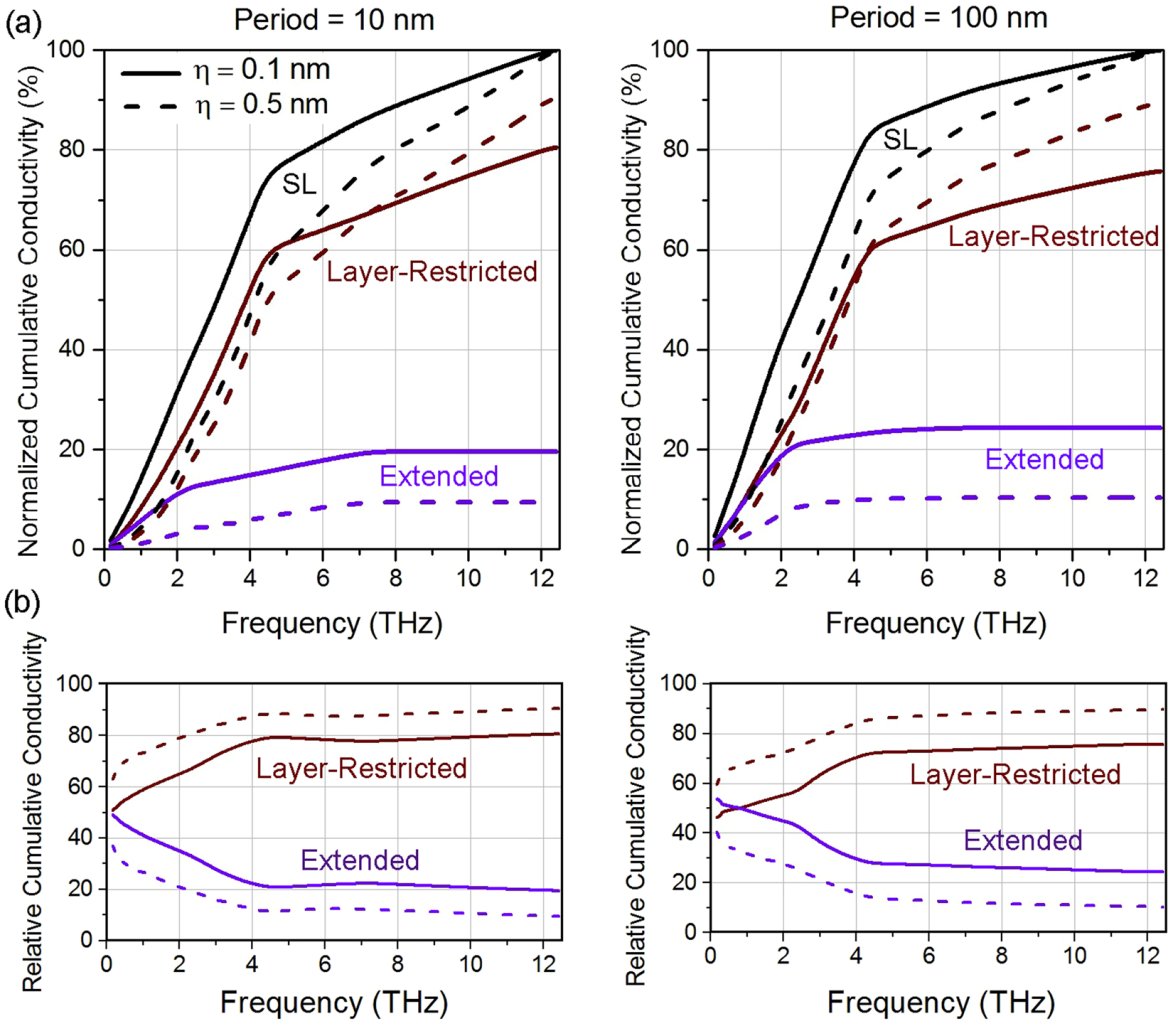
(**a**) Frequency spectrum for Si/Ge SL of period lengths *d* = 10 nm and *d* = 100 nm at roughness values of η = 0.1 nm and η = 0.5 nm (solid and dashed curves respectively). (**b**) Relative cumulative contribution of extended and layer-restricted phonons (purple and brown respectively) as a function of frequency in the corresponding SLs.

One important aspect in nanoscale heat transport is the establishment of the amount of heat carried by phonons with different frequencies. The prediction of the heat frequency spectrum allows to determine whether heat is carried by low, middle, or high frequency phonons, which can be utilized to rationally design thermal materials and devices^42–44^. Next, we study the heat frequency spectrum in Si/Ge superlattices. Figure 6 shows the normalized cumulative conductivity and relative cumulative conductivity at room temperature as a function of frequency for period lengths *d* = 10 nm and 100 nm, and *f*_Si_ = *f*_Ge_ = 0.5. Two different surface roughnesses were considered for both superlattices, *η* = 0.1 nm and 0.5 nm. Note that when the period decreases, the proportion of heat carried by high frequency phonons increases. For instance, for *d* = 10 nm, *η* = 0.1 nm, approximately 50% of the heat is carried by phonons with frequencies less than 3 THz, while this proportion is increased to 60% for *d* = 100 nm. This is due to reduced phonon scattering at the boundaries (due to lower interface density) in longer period length superlattices, which allows for larger mean-free-paths. Thus, a lower frequency phonon can carry more heat in a longer period superlattice. Figure 6a also quantitatively establishes the shift in the frequency spectrum with change in interface roughness. We note that with increase in roughness, the total superlattice conductivity spectrum shifts towards the right i.e. higher frequencies (or blue shift). This suggests that increase in interface roughness scatters lower frequencies more significantly (due to larger MFPs), thus higher frequencies carry more heat. The kink around 5 THz is due to the existence of only longitudinal modes at high frequencies. In Fig. 6b, we present the relative cumulative conductivity as a function of phonon frequency. The plots indicate the fraction of heat carried by layer-restricted and extended phonons up to a certain frequency for different periods and surfaces roughnesses. For example, at *d* = 100 and *η* = 0.5 nm, approximately 65% of the heat conduction is layer-restricted and the remaining ~35% is extended for phonons up to 3 THz in frequency. Figure 6b shows that with increasing interface roughness the fraction of heat that is layer-restricted is increased. This is consistent with the limiting case of fully diffuse interfaces wherein there will be no thermal conductivity contribution of extended modes and all heat will be layer-restricted. This observation provides a mechanism that can be utilized to control the proportion of thermal conductivity which is layer-restricted and extended by manipulating the roughness. We provide further verification of this trend in the next section. We also note that as frequency increases more phonons tend to be restricted. This is attributed to the larger mean-free-paths of low-frequency phonons which allow them to reach the interfaces to a larger extent. Also, low-frequency phonons have large wavelengths and are more specularly reflected at the interfaces, enabling transmission across different layers.

### The phonon mean-free-path spectrum

**Figure 7:**
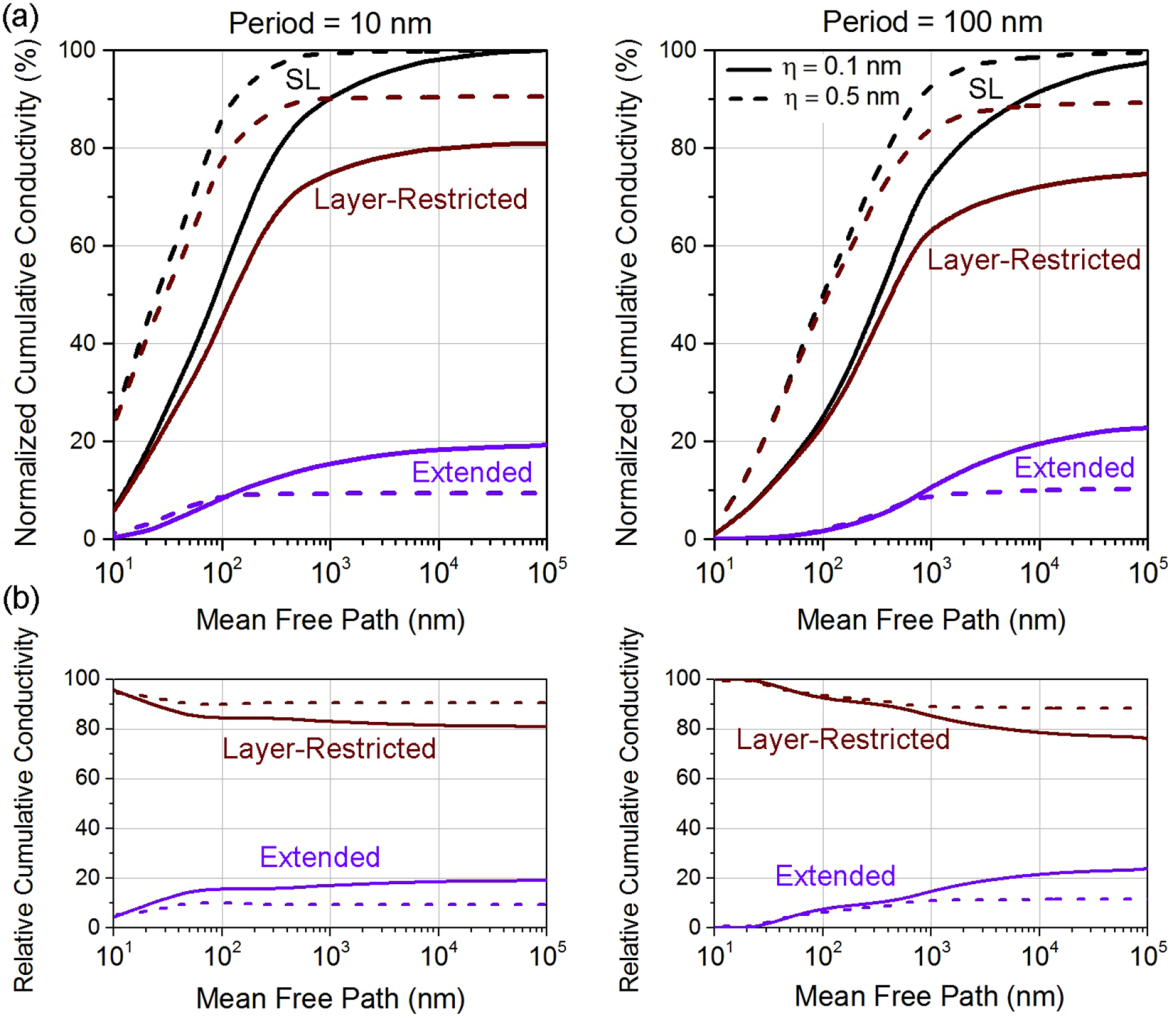
(**a**) Mean-free-path spectrum for Si/Ge SL of period lengths *d* = 10 nm and 100 nm at roughness values of 0.1 nm and 0.5 nm (solid and dashed curves respectively). (**b**) Relative cumulative contribution of extended and layer-restricted phonons (purple and brown respectively) as a function of mean-free-path in the corresponding SLs.

In addition to the frequency spectrum, a critical thermal transport property is the phonon mean-free-path spectrum, which allows to determine how far phonons move while carrying heat. In superlattices, the reduced phonon MFPs in each layer *i* depend on the *z*-coordinate of the point where the phonon originates and the wavevector $\mathop{k}\limits^{\longrightarrow}$, and are given by the solutions of the BTE (See Methods). We consider superlattices of period lengths 100 nm and 10 nm and surface roughness of 0.1 nm and 0.5 nm (*f*_Si_ = *f*_Ge_ = 0.50). Figure 7a gives the normalized cumulative conductivity at room temperature as a function of the MFP. The normalized cumulative conductivity tends to saturate starting at MFPs approximately 1 μm for period *d* = 10 nm. This implies that phonons having MFP larger than the saturation MFP do not carry significant portion of heat. We note that for all the components of conductivity, the saturation MFP shifts to higher values as the period length increases to *d* = 100 nm i.e. the curves shift to the right. This is consistent, since a shorter period length would imply shorter MFPs due to higher influence of boundary scattering. We observe that, in agreement with the frequency spectrum, more heat is layer-restricted than extended between the layers. This is due to the following reasons – (1) high frequency phonons of Si having frequency higher than the maximum frequency allowed in Ge (for either polarization – longitudinal or transverse) carry significant amount of heat and (2) phonons that are restricted in Ge via total internal reflection (due to the large acoustic impedance between Si and Ge) also carry substantial heat. Also, in agreement with the previous section, we observe that with increasing roughness, a larger fraction of heat conduction is restricted to a layer.

### Temperature dependence and comparison with experiments

**Figure 8:**
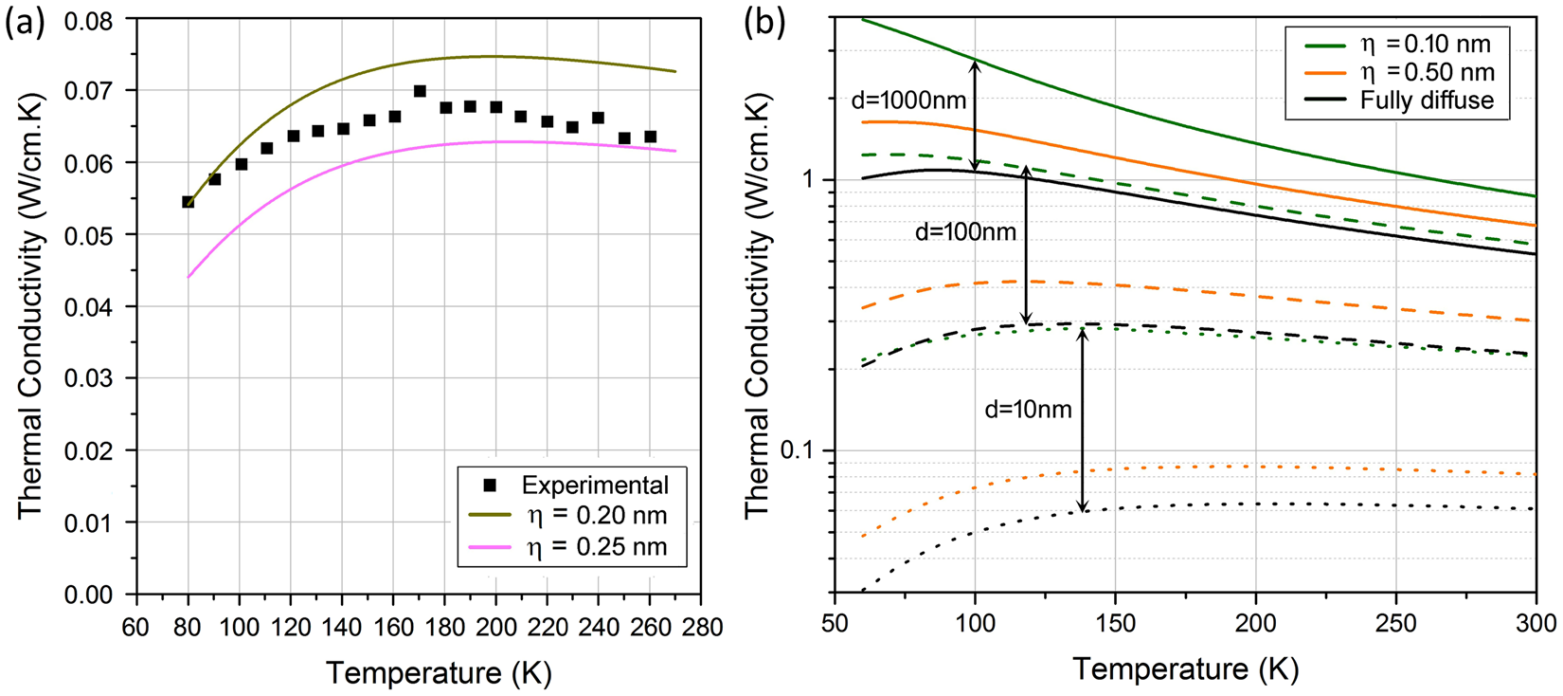
(**a**) Comparison between the theoretical predictions and experimental data for a 50-50 Si-Ge SL of period length 4 nm. (**b**) Temperature variation of thermal conductivity for Si-Ge SL of periods length *d* = 10 nm, 100 nm, and 1000 nm (dotted, dashed and solid curves respectively) at roughness values η = 0.1 nm, η = 0.5 nm and fully diffuse scattering (green, orange and black curves respectively).

Figure 8a shows the comparison between theoretical predictions and experimental measurements of superlattice thermal conductivity as a function of temperature. We note that measurements of in-plane thermal conductivity of SiGe superlattices in the literature are scarce. The theoretical (solid lines) and experimental (symbols) plots show the temperature variation of in-plane thermal conductivity in a Si/Ge superlattice with period *d* = 4 nm and *f*_Si_ = *f*_Ge_ = 0.50. We observe that κ_SL_ initially increases with increase in temperature, reaches a maximum and then shows a weak dependence with increase in temperature. The observed reduction of κ_SL_ at low temperatures is a result of decreasing phonon occupation as the temperature is reduced. At higher temperatures, phonon occupation does not change significantly, and the weak dependence of κ_SL_ on temperature is a consequence of the dominance of interface scattering over phonon-phonon scattering due to the small superlattice period. We see that our predictions can explain the trends by providing a match with the experimental data^35^. In addition, Fig. 8a shows the sensitivity of κ_SL_ with respect to surface roughness where a small decrease in *η* leads to a slight increase in κ_SL_. We provide in Fig. 8b the dependence of κ_SL_ on temperature for a vast range of structural features, where periods are increased by orders of magnitude from *d* = 10 nm to *d* = 1μm, and three surface roughness conditions *η* = 0.1 nm, *η* = 0.5 nm, and fully diffusive interfaces are considered. Note that at high temperatures (>100 K), with increasing period length, there is a reduction of thermal conductivity with temperature due to phonon-phonon scattering, in contrast to a nearly constant dependence for small periods due to the dominance of phonon-surface scattering.

### Phonon wave effects

In this section, we analyze the possibility of wave effects, which is an emerging research area that has captured significant interest in recent years. For certain structures and temperatures, wave effects can modify phonon dispersion relations, opening opportunities to control phonon group velocities and density of states, which are critical transport properties affecting the thermal conductivity^10^. The coherent interference of multiple reflected phonons at interfaces is the basic mechanism for the appearance of wave effects. It is important to highlight that thermal phonons are incoherent in the sense that they are formed by random vibrations of the atomic lattice. Although thermal phonons are incoherently generated, wave effects due to coherent interference refer to wave effects arising from the coherent interference of phonons with themselves after reflection and transmission at multiple surfaces or interfaces. In what follows, we explain why wave effects are difficult to obtain for room temperature in-plane thermal transport in Si/Ge superlattices and provide guidelines to achieve wave effects for thermal phonons in superlattices. There exist two wave effects that can develop in superlattices: (1) *for layer-restricted phonons*, multiple reflections at the surface boundaries of the layer can interfere coherently and given rise to “quantum confinement” effects; (2) *for extended phonons*, multiple reflections at the interfaces between the layers of the superlattice can interfere coherently and give rise to “phononic band gaps”. We note that in both cases the net effect is the modification of the phonon dispersion relations and thus the velocities and density of states of thermal phonons. The specific conditions for development of phonon quantum confinement effects in terms of film thickness, roughness and temperatures have been analyzed in ref. 45. In this paper, we focus our analysis on the development of coherent interference for extended phonons.

Extended phonons propagate through different layers in the superlattice. Figures 2, 3, 4 and 5 show that the amount of heat they carry is dependent on the superlattice structural features and in general is smaller than that carried by layer-restricted phonons. For period *d* = 100 nm, roughness η = 0.1 nm, and *f*_Si_ = 0.75 − *f*_Ge_ = 0.25, however, the relative amount of heat carried by extended phonons is close to 30%. This is therefore the most favorable superlattice for the development of coherent interference in the sense that 30% of the heat is carried by phonons that see multiple interfaces. We note, however, that for strong coherent interference effects in SiGe phonons must see at least ten interfaces^46, 47^. We calculated the fraction of heat carried by phonons that travel through five superlattice periods (i.e. 10 interfaces) and found that it is reduced to 16%. This small amount is one reason why strong wave effects and a minimal thermal conductivity are not observed for in-plane transport. We would like to clarify that bulk phonon mean free paths in Si and Ge can be relatively long but they are strongly reduced in superlattices due to surface scattering. As a result, only a reduced fraction of phonons can travel across multiple layers when the Si-Ge superlattice interfaces are rough. Since coherent interference of extended phonons can lead to wave effects, having a small fraction of those, leads to negligible wave effects and bulk material parameters can therefore be used. This is also in agreement with the literature as strong wave effects have been demonstrated only for cross-plane phonon transport with smooth interfaces. In fact, in-plane phonon transport is not the ideal case since phonons that see the largest number of interfaces (before thermalizing) propagate normally to the temperature gradient and therefore do not conduct a significant amount of heat. Although coherent interference effects for in-plane phonon transport in Si/Ge superlattices at room temperature are small, phonon wave effects can be achieved by releasing the constraints as follows. *Cross-plane phonon transport:* When the temperature gradient ∇*T* is normal to the layers, layer-restricted phonons travel perpendicularly to ∇*T* while extended phonons (which can be subject to coherent interference) move along ∇*T*. This is in direct contrast to in-plane phonon transport. As a result, for cross-plane phonon transport, phonons that can be subject to coherent interference carry a more significant part of the heat, and wave effects may become significant. Cross-plane phonon wave effects have been reported theoretically using wave theory^12^ and molecular dynamics^18, 19^ and experimentally in oxide superlattices^32^. *Alloying:* In alloys, the mass-difference in the atomic lattice gives rise to diffuse scattering of short-wavelength phonons, restricting their ability to carry heat^43, 44, 48^. As a result, the relative amount of heat carried by long-wavelength phonons increases with respect to the pure materials. Phonons with long wavelengths (i.e. low frequencies) have large mean-free-paths and are more prone to undergo specular interface scattering, which increases the likelihood of developing extended modes and wave effects^10^. *Low temperature:* At low temperatures, the proportion of heat carried by long-wavelength phonons increases but for reasons different to those in alloying. In this case, high-frequency phonons are not thermally excited and therefore do not carry heat. The heat spectrum is thus made of long-wavelength phonons, which are likely to scatter specularly at interfaces and develop extended modes and wave effects. Wave effects at low temperatures have been recently demonstrated experimentally in two-dimensional periodic porous materials^49, 50^. It is important to highlight that wave effects would appear for certain structures under certain conditions, and full characterization of the structures in terms of periodicity *and* interface roughness as well as temperature needs to be investigated to clearly establish when wave effects on thermal transport can develop in engineered nanosystems.

## Summary

In summary, we studied thermal phonon transport in Si/Ge superlattices using a rigorous Boltzmann transport model that includes the effects of phonon coupling between layers, superlattice periodicity, and interface surface conditions while also considering the dependence of surface specularity on phonon wavelength, angle of incidence, and roughness. Our model thus incorporates all relevant physical properties relevant to heat conduction in superlattices. We provided a detailed description of the amount of thermal energy carried by phonons restricted to a layer and those which are extended over multiple layers in the superlattice. We predicted the variation of thermal conductivity with varying period length, roughness and volume fraction for both layer-restricted and extended phonons. The results provide new tools to manipulate the proportion of heat that is conducted in a single or multiple layers and can be utilized for the rational design of thermal systems. For in-plane phonon transport at room temperature we found that, in general, heat is primarily carried by phonons restricted within the layers. Based on the detailed model developed, we also computed the frequency and mean-free-path spectrum for superlattices of different period lengths and surface conditions. The frequency and MFP spectra can be utilized to design thermal devices based on superlattices as material platforms, as they allow to determine ballistic vs. diffusive and coherent vs. incoherent heat conduction regimes. Wave effects for Si/Ge in-plane phonon transport at room temperature were found to be small but they can be increased by considering cross-plane transport, alloying or low temperatures. The fundamental understanding of thermal phonon transport processes in superlattices holds the promise of rational design of thermal materials and devices with unprecedented heat flux control.

## Methods

### Thermal conductivity

The in-plane thermal conductivity of superlattices is calculated by using the well-established Fourier’s law of heat conduction, which is given by

1 $$\mathop{j}\limits^{\longrightarrow}=-\kappa \mathop{\nabla }\limits^{\longrightarrow}T$$

where $\mathop{j}\limits^{\longrightarrow}$ is the heat flux, *κ* is thermal conductivity and $\mathop{\nabla }\limits^{\longrightarrow}T$ is the temperature gradient. Kinetic theory establishes that the heat flux^4^ $\mathop{j}\limits^{\longrightarrow}$ is given by the product of the energy $\hslash \omega$ carried by phonons, their distribution functions *f* and their velocities $\mathop{v}\limits^{\longrightarrow}$. Integration over all possible phonons with wavevectors $\mathop{k}\limits^{\longrightarrow}$ gives the thermal flux as

2 $$\mathop{j}\limits^{\longrightarrow}=\frac{1}{{(2\pi )}^{3}}\sum _{p}\int \hslash {\omega }_{\mathop{k}\limits^{\longrightarrow},p}{f}_{\mathop{k}\limits^{\longrightarrow},p}{\mathop{v}\limits^{\longrightarrow}}_{\vec{k},p}{d}^{3}k$$

where the subscript *p* denotes the different polarizations of phonons (i.e. longitudinal and transverse). The thermal conductivity *κ* of the superlattice is calculated by combining Eqs (1) and (2) and integrating the flux along the superlattice period *t*_0_ as

3 $$-\kappa \mathop{\nabla }\limits^{\longrightarrow}T=\frac{1}{{t}_{0}}\int \mathop{j}\limits^{\longrightarrow}dl$$

The calculation of the thermal conductivity using Eqs (2) and (3) requires the distribution of phonons ${f}_{\mathop{k}\limits^{\longrightarrow},p}$ for the superlattice, which is determined by the Boltzmann Transport Equation (BTE).

### Boltzmann transport equation

The generalized form of the Boltzmann Transport Equation, which allows to obtain the phonon distribution functions ${f}_{\mathop{k}\limits^{\longrightarrow},p}$, is given by ref. 4

4 $$\frac{\partial {f}_{\mathop{k}\limits^{\longrightarrow},p}}{\partial t}+\,{\mathop{v}\limits^{\longrightarrow}}_{\mathop{k}\limits^{\longrightarrow},p}.\mathop{\nabla }\limits^{\longrightarrow}{f}_{\mathop{k}\limits^{\longrightarrow},p}={(\frac{\partial {f}_{\mathop{k}\limits^{\longrightarrow},p}}{\partial t})}^{scatt}$$

Since we are interested in solving the BTE for phonons in the steady-state case, i.e. constant heat flux, the first term vanishes. Using the single-mode relaxation time approximation^4^, the scattering term on the right-hand side of Eq. (4) is given by $\frac{{f}^{0}-f}{\tau }$, where *f* ^0^ is the equilibrium phonon distribution function given by the Bose-Einstein distribution function and $\tau =\tau (\mathop{k}\limits^{\longrightarrow},p,T)$ is the phonon relaxation time, which is a function of the wavevector $\mathop{k}\limits^{\longrightarrow}$, polarization *p*, and temperature *T*. Given that the above equation is applicable for each polarization *p* and each $\mathop{k}\limits^{\longrightarrow}$-mode, subscripts will be omitted. Expanding *f* = *f* ^0^ + *g*, where *g* is the deviation function from the equilibrium, Eq. (4) reduces to

5 $$\mathop{v}\limits^{\longrightarrow}.\mathop{\nabla }\limits^{\longrightarrow}f=-\frac{g}{\tau }$$

In general *f* is a function of the position vector $\mathop{r}\limits^{\longrightarrow}$. We define our coordinate system such that the superlattice is periodic in the *z*-direction, while being uniform in the *x* and *y* directions (Fig. 1). The thermal gradient is applied in the *x*-direction so the equilibrium distribution *f* ^0^ is dependent only on the *x*-coordinate and the distribution *f* is independent of the *y* coordinate due to continuous translation symmetry. Assuming that the deviation from equilibrium is small, i.e. *g* ≪ *f* ^0^, we can neglect the *x*-derivative of *g* and transform Eq. (5) into

6 $${v}_{x}\frac{\partial {f}^{0}}{\partial T}\frac{\partial T}{\partial x}+{v}_{z}\frac{\partial g}{\partial z}=-\frac{g}{\tau }$$

By considering the general solution of the differential equation (6), *g* can be written as a function of the *z* coordinate and an arbitrary function ${\phi }_{\mathop{k}\limits^{\longrightarrow}}$

7 $${g}_{\mathop{k}\limits^{\longrightarrow }}(z)=-{v}_{x}\tau \frac{{\rm{\partial }}{f}^{0}}{{\rm{\partial }}T}\frac{{\rm{\partial }}T}{{\rm{\partial }}x}[1+{\phi }_{\mathop{k}\limits^{\longrightarrow }}\,\exp (\frac{-z}{{v}_{z}\tau })]$$

The function ${\phi }_{\mathop{k}\limits^{\longrightarrow}}$ determining the phonon distribution function is rigorously calculated in the next section by applying the boundary conditions of the superlattice, which are implemented after a careful analysis of phonon surface scattering at the interfaces. Once ${\phi }_{\mathop{k}\limits^{\longrightarrow}}$ (and therefore ${g}_{\mathop{k}\limits^{\longrightarrow}}$) is calculated, Eqs (1)–(3) allow to obtain the resultant thermal conductivity of the superlattice.

### Phonon scattering at rough surfaces

For infinitely periodic superlattices, the boundary conditions are given by an energy balance upon scattering of phonons at the rough interfaces. We use a rigorous statistical analysis to study the scattering of thermal phonons from rough surfaces. The scattering of electromagnetic waves from rough surfaces has been studied in detail by Beckmann and Spizzichino^36^. This analysis however is limited to free surfaces (e.g. solid-air interfaces) preventing the study of phonon scattering at surfaces between two different solid materials. We employ a generalized analysis of surface scattering^51^, where the Beckmann-Kirchhoff theory has been extended to include forward scattering i.e. the reflection and transmission of phonons at a rough interface between two homogeneous and isotropic solids. We outline below the main points to obtain a generalized solution for scattering between two interfaces and subsequently extend it to the case of superlattices.

Consider the height *z* of an interface between two media 1 and 2 (with respect to a middle plane) given by the function *z* = *ζ*(*x*, *y*), where the average value of *z*, i.e. 〈*ζ*(*x*, *y*)〉 with respect to the plane is zero. The region for media 1 is given by *z* > *ζ*(*x*, *y*) and that for media 2 is given by *z* < *ζ*(*x*, *y*). Assuming that *ζ* is a random variable representing the heights of the surface, the roughness of the surface *η* is defined as the standard deviation of *ζ*. Without losing generality, we assume that the incident wavevector $\mathop{k}\limits^{\longrightarrow}$ lies in the *x*-*z* plane. To describe the interaction of phonons with an interface, consider ${\rm{u}}=\exp [i(\mathop{k}\limits^{\longrightarrow}.\mathop{r}\limits^{\longrightarrow})-\omega t]$ be the displacement phonon field which is the solution of the Helmholtz equation Δ*u* + *k* ^2^ *u* = 0^36^. When the radius of curvature of the interface is much larger than the wavelength, the Kirchhoff boundary conditions apply^36^

8 $$u{|}_{z \rightarrow {\zeta }^{+}}=(1+\Re ){e}^{i\mathop{k}\limits^{\longrightarrow}.{\mathop{r}\limits^{\longrightarrow}}_{s}},\quad \frac{\partial u}{\partial n}{|}_{z \rightarrow {\zeta }^{+}}=i(1-\Re )(\mathop{k}\limits^{\longrightarrow}.\,\mathop{n}\limits^{\longrightarrow}){e}^{i\mathop{k}\limits^{\longrightarrow}.{\mathop{r}\limits^{\longrightarrow}}_{s}}$$

9 $$u{|}_{z \rightarrow {\zeta }^{-}}=\Im {e}^{i\mathop{k}\limits^{\longrightarrow}.\mathop{{r}_{s}}\limits^{\longrightarrow}},\quad \frac{\partial u}{\partial n}{|}_{z \rightarrow {\zeta }^{-}}=i\Im (\mathop{\tilde{k}}\limits^{\longrightarrow}.\,\mathop{n}\limits^{\longrightarrow}){e}^{i\vec{k}.\mathop{{r}_{s}}\limits^{\longrightarrow}}$$

where $\mathop{n}\limits^{\longrightarrow}$ denotes the unit vector normal to the surface *z* = *ζ*(*x*, *y*) at point $\overrightarrow{{r}_{s}}=x\widehat{x\,}+y\hat{y}+\,\zeta (x,y)\hat{z}$ and $\mathop{\tilde{k}}\limits^{\longrightarrow}$ is the local refracted wave vector. Also, $\Re$ and $\Im$ denote the standard reflection and transmission coefficients from a *perfectly smooth* surface, respectively. The solution of the Helmholtz equation at a distance *R* from the surface, at $\mathop{R^{\prime} }\limits^{\longrightarrow}$ and $\mathop{R^{\prime\prime} }\limits^{\longrightarrow}$ in medium 1 and 2, respectively, gives

10 $$u(\mathop{R^{\prime} }\limits^{\longrightarrow})\approx \,\frac{i{e}^{i{k}_{1}R}}{4\pi R}{\int }_{S}[\Re (\mathop{k}\limits^{\longrightarrow}-\mathop{k^{\prime} }\limits^{\longrightarrow})-(\mathop{k}\limits^{\longrightarrow}+\mathop{k^{\prime} }\limits^{\longrightarrow})]\cdot \,\vec{n}{e}^{i(\mathop{k}\limits^{\longrightarrow}-\mathop{k^{\prime} }\limits^{\longrightarrow}).\mathop{{r}_{s}}\limits^{\longrightarrow}}dS$$

11 $$u(\mathop{R^{\prime\prime} }\limits^{\longrightarrow})\approx \,\frac{i{e}^{i{k}_{2}R}}{4\pi R}{\int }_{S}\Im (\mathop{\tilde{k}}\limits^{\longrightarrow}+\overrightarrow{k^{\prime\prime} })\cdot \vec{n}{e}^{i(\mathop{k}\limits^{\longrightarrow}-\mathop{k^{\prime\prime} }\limits^{\longrightarrow}).\mathop{{r}_{s}}\limits^{\longrightarrow}}dS$$

where $\mathop{k^{\prime} }\limits^{\longrightarrow}$ is the reflected wave vector, $\mathop{k^{\prime\prime} }\limits^{\longrightarrow}$ is the overall reflected wave vector, *k*_1_ is the magnitude of the incident and reflected wavevector, and *k*_2_ is the magnitude of the refracted wave vector. Carrying out a rigorous statistical analysis^51^, one can obtain the reflection *P*_*ij*_ and transmission *Q*_*ij*_ coefficients from a *rough* interface, where *i* and *j* denote the two solid media across the surface, which are given by

12 $${P}_{ij}={\Re }_{ij}^{2}\,\exp (-4{\eta }^{2}{k}_{i}^{2}{\cos }^{2}{\theta }_{i})$$

13 $${Q}_{ij}=(1-{\Re }_{ij}^{2})\exp (-{\eta }^{2}{({k}_{i}\cos {\theta }_{i}-{k}_{j}\cos {\theta }_{j})}^{2})$$

where

14 $${\Re }_{ij}^{2}=\,{(\frac{{\rho }_{i}{v}_{i}\cos {\theta }_{i}-{\rho }_{j}{v}_{j}\cos {\theta }_{j}}{{\rho }_{i}{v}_{i}\cos {\theta }_{i}+{\rho }_{j}{v}_{j}\cos {\theta }_{j}})}^{2}$$

In these equations, *ρ*_*i*_, *θ*_*i*_, and *v*_*i*_ denote density (*ρ*_*Si*_ = 2.33 g/cm^3^, *ρ*_*Ge*_ = 5.32 g/cm^3^), incident angle, and group velocity *ν*_*i*_ = ∇_**k**_ *ω*_*i*_(**k**), where *ω*_*i*_(**k**) is the phonon dispersion relation in medium *i*, respectively. Since ${\Re }_{ij}^{2}={\Re }_{ji}^{2}$, we have *Q*_*ij*_ = *Q*_*ji*_, which is the principle of detailed balance for phonons. Independent of the nature of the boundary conditions, the law of reflection and refraction states that the tangential component of the wavevector is conserved. That is

15 $${k}_{i}\,\sin \,{\theta }_{i}={k}_{j}\,\sin \,{\theta }_{j}$$

For incident angles larger that the critical angle, ${\Re }_{ij}^{2}=1$ and phonons are subject to total internal reflection and therefore restricted to propagate within a layer. Importantly, the surface scattering model described above thus allows to obtain the phonon thermal energy reflected and transmitted at *rough* interfaces in superlattices.

### Boundary conditions and energy balance at interfaces

**Figure 9:**
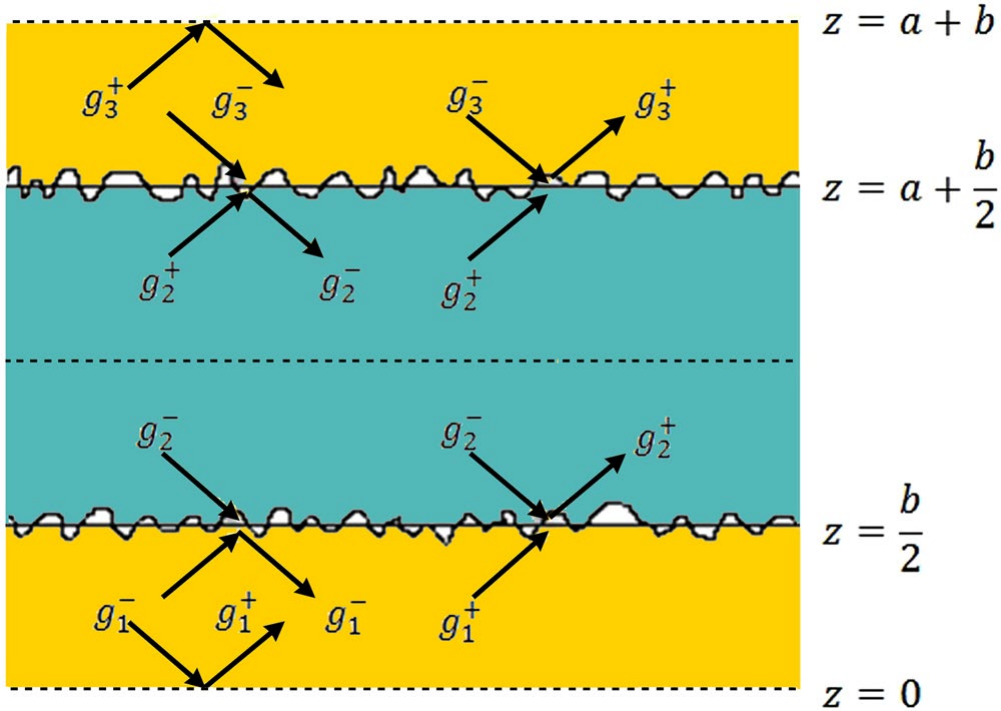
Diagram for the unit cell of a superlattice with rough interfaces and period *d* = *a* + *b*, where *a* and *b* are the thicknesses of the layers along the *z* direction. The symbols *g* correspond to the deviation functions from equilibrium for phonons propagating with wavevectors *k*_*z*_ along the +*z* direction (+) and −*z* direction (−) in the three superlattice regions: (1) 0 < *z* < *b*/2, (2) *b*/2 < *z* < *a* + *b*/2, and (3) *a* + *b*/2 < *z* < *a* + *b*.

Due to translational symmetry along the *z*-direction, the BTE needs to be solved only within a single unit cell of the superlattice, which when repeated in the *z*-direction reproduces the entire superlattice. In our calculations, the unit cell begins halfway through a layer of material B and extends halfway into the next layer of material B as illustrated in Fig. 9.

We write the general solution of *g* given by Eq. (7) as follows^52^

16 $${g}_{i}^{\pm }=-{v}_{x,i}{\tau }_{i}\frac{{\rm{\partial }}{f}_{i}^{0}}{{\rm{\partial }}T}\frac{{\rm{\partial }}T}{{\rm{\partial }}x}[1+{\phi }_{i}^{\pm }(\mathop{k}\limits^{\longrightarrow })\,\exp (\frac{\mp z}{|{v}_{z,i}|{\tau }_{i}})]$$

where the subscript *i* denotes a particular layer in the superlattice and the superscript ‘+’ or ‘−’ denotes the direction of *k*_*z*_, where ‘+’ denoted a positive *k*_*z*_ and vice versa. We define *l*_*x*_,_*i*_ = *v*_*x*,*i*_ *τ*_*i*_ and *l*_*z*,*i*_ = |*v*_*z*,*i*_|*τ*_*i*_. To exploit the translational symmetry of the superlattice, we consider (for each layer) translated versions of the general solution of *g*

$$\begin{array}{rcl}{g}_{1}^{+} & = & -{l}_{x,1}\frac{\partial {f}_{1}^{0}}{\partial T}\frac{\partial T}{\partial x}[1+\,{\phi }_{1}^{+}(\mathop{{k}}\limits^{\longrightarrow})\exp (-\frac{z+(b/2)}{{l}_{z,1}})]\\ {g}_{1}^{-} & = & -{l}_{x,1}\frac{\partial {f}_{1}^{0}}{\partial T}\frac{\partial T}{\partial x}[1+\,{\phi }_{1}^{-}(\mathop{{k}}\limits^{\longrightarrow})\exp (+\frac{z-(b/2)}{{l}_{z,1}})]\\ {g}_{2}^{+} & = & \,-{l}_{x,2}\frac{\partial {f}_{2}^{0}}{\partial T}\frac{\partial T}{\partial x}[1+\,{\phi }_{2}^{+}(\mathop{{k}}\limits^{\longrightarrow})\exp (-\frac{z-(b/2)}{{l}_{z,2}})]\\ {g}_{2}^{-} & = & -{l}_{x,2}\frac{\partial {f}_{2}^{0}}{\partial T}\frac{\partial T}{\partial x}[1+\,{\phi }_{2}^{-}(\mathop{{k}}\limits^{\longrightarrow})\exp (+\frac{z-(a+(b/2))}{{l}_{z,2}})]\\ {g}_{3}^{+} & = & -{l}_{x,3}\frac{\partial {f}_{3}^{0}}{\partial T}\frac{\partial T}{\partial x}[1+\,{\phi }_{3}^{+}(\mathop{{k}}\limits^{\longrightarrow})\exp (-\frac{z-(a+(b/2))}{{l}_{z,3}})]\\ {g}_{3}^{-} & = & -{l}_{x,3}\frac{\partial {f}_{3}^{0}}{\partial T}\frac{\partial T}{\partial x}[1+\,{\phi }_{3}^{-}(\mathop{{k}}\limits^{\longrightarrow})\exp (+\frac{z-(a+(3b/2))}{{l}_{z,3}})]\end{array}$$

Note that since the phonon frequency remains constant during reflection and transmission, we can simplify the expressions in Eq. (17) and omit the term $\frac{\partial {f}_{i}^{0}}{\partial T}\frac{\partial T}{\partial x}$ when applying the boundary conditions. By considering the mirror symmetries, the following boundary conditions apply for the superlattice^52^

18 $${g}_{1}^{+}=\,{g}_{1}^{-}\,at\,z=0$$

19 $${g}_{3}^{+}={g}_{3}^{-}\,at\,z=a+b$$

In addition, at the interfaces between different materials, energy balance for phonon reflection and transmission establishes that

20 $${g}_{1}^{-}(-{k}_{z})={P}_{12}\,{g}_{1}^{+}({k}_{z})+{Q}_{12}{g}_{2}^{-}(-{k}_{z}^{^{\prime} })\quad atz=b/2$$

21 $${g}_{2}^{+}({k}_{z}^{^{\prime} })={P}_{21}{g}_{2}^{-}(-{k}_{z}^{^{\prime} })+{Q}_{12}{g}_{1}^{+}({k}_{z})\quad atz=b/2$$

22 $${g}_{2}^{-}(-{k}_{z}^{^{\prime} })={P}_{23}{g}_{2}^{+}({k}_{z}^{^{\prime} })+{Q}_{23}{g}_{3}^{-}(-{k}_{z})\quad atz=a+b/2$$

23 $${g}_{3}^{+}({k}_{z})={P}_{32}{g}_{3}^{-}(-{k}_{z})+{Q}_{23}{g}_{2}^{+}({k}_{z}^{^{\prime} })\quad atz=a+b/2$$

Note that the primed notation is to emphasize that while in medium 1 the wavevector is $\mathop{k}\limits^{\longrightarrow}$, the wavevector in medium 2 is $\mathop{k^{\prime} }\limits^{\longrightarrow}$. The solution of the system of Eqs (20)–(23) fully determines the functions ${\phi }_{i}^{\pm }(\mathop{k}\limits^{\longrightarrow})$ which establish the distribution of phonons ${g}_{i}^{\pm }$ deviated from equilibrium for each layer [Eq. (17)]. Calculation of the distribution functions ${g}_{i}^{\pm }$ allows us to determine the thermal conductivity using Eqs (2) and (3). Note that all variables in our theoretical framework depend on the wavevector $\mathop{k}\limits^{\longrightarrow}$, allowing to perform a full frequency-dependent analysis of all phonon transport properties, including a rigorous treatment of phonon scattering at the interfaces and phonon coupling between superlattice layers.
